# Impacts on the Deep-Sea Ecosystem by a Severe Coastal Storm

**DOI:** 10.1371/journal.pone.0030395

**Published:** 2012-01-25

**Authors:** Anna Sanchez-Vidal, Miquel Canals, Antoni M. Calafat, Galderic Lastras, Rut Pedrosa-Pàmies, Melisa Menéndez, Raúl Medina, Joan B. Company, Bernat Hereu, Javier Romero, Teresa Alcoverro

**Affiliations:** 1 Grup de Recerca Consilidat (GRC)-Geociències Marines, Departament d'Estratigrafia, Paleontologia i Geociències Marines, Universitat de Barcelona, Barcelona, Spain; 2 Instituto de Hidraúlica Ambiental “IH Cantabria”, Universidad de Cantabria, Santander, Spain; 3 Institut de Ciències del Mar, Consejo Superior de Investigacions Cientificas (CSIC), Barcelona, Spain; 4 Departament d'Ecologia, Universitat de Barcelona, Barcelona, Spain; 5 Centre d'Estudis Avançats de Blanes, Consejo Superior de Investigacions Cientificas (CSIC), Blanes, Spain; University of California, Merced, United States of America

## Abstract

Major coastal storms, associated with strong winds, high waves and intensified currents, and occasionally with heavy rains and flash floods, are mostly known because of the serious damage they can cause along the shoreline and the threats they pose to navigation. However, there is a profound lack of knowledge on the deep-sea impacts of severe coastal storms. Concurrent measurements of key parameters along the coast and in the deep-sea are extremely rare. Here we present a unique data set showing how one of the most extreme coastal storms of the last decades lashing the Western Mediterranean Sea rapidly impacted the deep-sea ecosystem. The storm peaked the 26^th^ of December 2008 leading to the remobilization of a shallow-water reservoir of marine organic carbon associated with fine particles and resulting in its redistribution across the deep basin. The storm also initiated the movement of large amounts of coarse shelf sediment, which abraded and buried benthic communities. Our findings demonstrate, first, that severe coastal storms are highly efficient in transporting organic carbon from shallow water to deep water, thus contributing to its sequestration and, second, that natural, intermittent atmospheric drivers sensitive to global climate change have the potential to tremendously impact the largest and least known ecosystem on Earth, the deep-sea ecosystem.

## Introduction

**Figure 1:**
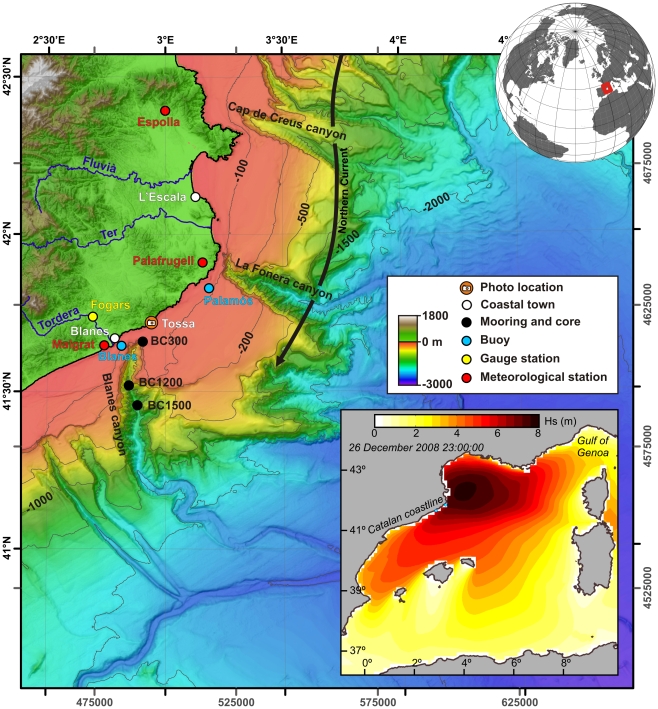
Map of the study area. Bathymetric map of the Catalan margin and station location including the moorings and sediment core stations (black dots), wave buoys (blue dots), meteorological stations (red dots), gauging station (yellow dot) and Figure 3 photo location (orange dot). Arrow indicates the direction of the Northern Current. The inset shows the spatial variability of the Hs (m) calculated with an atmospheric model in the Western Mediterranean Sea. See also Fig. S1 and Supporting Methods section.

Extreme events are rare opportunities of high scientific value that allow us to investigate how natural processes at their peaks of activity transfer matter and energy within and across ecosystem boundaries, and to identify the mechanisms involved and their rates, jointly with their final ecosystemic impact. The Mediterranean Sea is one of the most cyclogenic regions in the northern hemisphere during winter time, when episodes of extreme weather are common. The highest frequency of wind storms occurs in its northwestern basin [1], and is associated to E-NE and NW intense atmospheric fluxes. The general NE-SW orientation of the Catalan coastline in the northwestern Mediterranean Sea results in about 1,000 km fetch for strong, persistent E-NE winds generating high waves over a 0.8 to 30 km wide continental shelf, being narrowest where it is carved by large submarine canyon heads (Fig. 1) [2], [3]. Such atmospheric forcing drives shallow oceanographic processes at various spatial and temporal scales and interferes with shelf sediment transport patterns [4], [5], also causing recurrent beach erosion, overwash and inundation of low-lying areas along the shoreline.

Efforts to understand the deep sea environmental implications of such extreme weather perturbations have been hampered by the lack of concurrent measurements of key parameters such as near bottom flows or sediment characteristics. In this paper we provide evidence to show that a severe coastal storm recorded in 2008 in the Western Mediterranean impacted the deep sea ecosystem. Current meter and sediment trap data were collected during the passage of the storm, and we compare them with sediment cores collected prior to and after the storm. With these data, we investigate the impact of the storm on sediment erosion, transport and deposition from the coast to the deep sea, and its implications for understanding the organic carbon cycle.

## Results and Discussion

**Figure 2:**
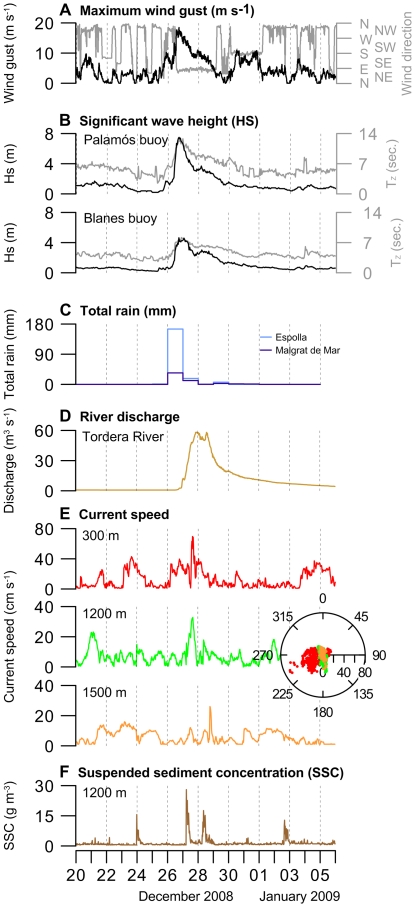
Time series of key parameters. **A**, Maximum wind gust (m s^−1^) and direction as observed in the meteorological station of Palafrugell; **B**, Significant wave height (Hs, black line) and mean period (Tz, grey line) in the Palamós and Blanes buoys; **C**, Total precipitation in Malgrat de Mar and Espolla meteorological stations; **D**, Tordera river discharge; **E**, Current speed at 300 m, 1200 m and 1500 m water depth along the Blanes canyon axis; and **F**, suspended sediment concentration (SSC) at 1200 m depth. See locations in Figure 1. The marine response to the wind-generated storm was instantaneous, with a downcanyon current flow occurring almost simultaneously with the increased wave height. Decreasing scattering of current direction with depth confirms the along canyon constrainment of the current, and increasing SCC reflects spreading of particles to the deep after each current pulse at the canyon head.

A detailed analysis of wave time series allowed to identify the largest storms impacting the Catalan coast in the last 25 years. Severe storms are here defined as events in which the significant wave height exceeds a threshold value of 4.3 m, which corresponds to categories 4–5 of a five-class scale analogue to extreme event classifications according to [6]. Amidst 16 severe storms found (Fig. S1), the December 2008 case is the most extreme, with a return period in excess of 100 years [7]. Storms of equivalent intensity have been reported in the same area from early and mid twentieth century but there are no instrumental wave records of them. The storm under investigation formed on December 25, when a strong high pressure system reaching up to 1044 hPa over northeastern Europe blocked the western atmospheric circulation and led to northern cold air fluxes and a deep cyclone to develop in the northwestern Mediterranean Basin (Video S1). This favored maritime eastern winds blowing over the Catalan coast to generate stormy seas. The storm intensified to category 5 as it moved from the Gulf of Genoa to the Catalan coast, where it made landfall on December 26, 2008, with maximum winds up to 20 m s^−1^, significant wave heights as large as 8 m, record maximum wave heights in excess of 14 m, and wave periods of up to 14 s. The storm was followed by heavy rainfall in the entire northwestern Mediterranean area, causing riverine floods on December 28 (Fig. 2A, B, C, D).

**Figure 3:**
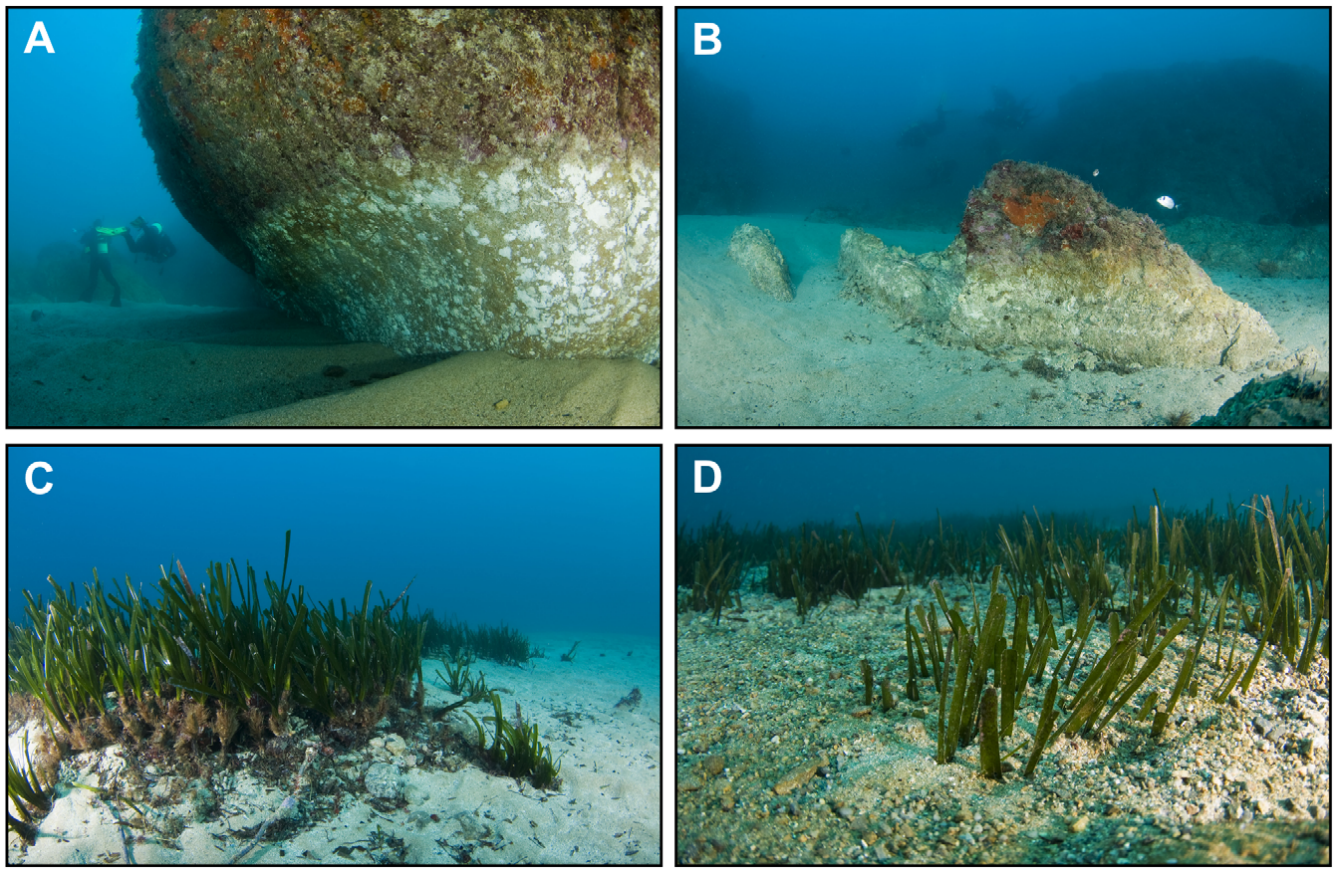
Photographs of the impact of the coastal storm in shallow water. **A** and **B**, loss of approx. 1 m of sandy sediments (see diver in A for scale). Change to lighter colour on the rocks indicates new exposure. **C**, unearthed shoots (foreground, dark-brown parts at the base of green leaves) and roots (dark-brown mass of rhizomes below and ahead of unearthed shoots) left after the storm at the location of a former *Posidonia oceanica* seagrass meadow (length of tallest plants including rhizomes is about 30 cm). Uprooting of *P. oceanica* because of the storm has been reported in the area. When plants have their rhizomes exposed, they are more vulnerable to abrasion, breaking and uprooting (as probably occurred to the missing shoots) by hydrodynamic forces and coarse sediment in motion. **D**, burial of a *Posidonia oceanica* meadow at 8–12 m water depth near Tossa de Mar (length of tallest leaves is about 12 cm) (credits for photographs: Jordi Regàs). See photo location in Figure 1.

Damage to shallow inner continental shelf benthic communities by currents and sand scouring during the storm is well documented after scientific scuba diving inspections. In rocky substrates, algal cover and population of sea urchins nearly disappeared by abrasion [8]. 80% mortality rates of the endemic and threatened Mediterranean brown algae *Cystoseira zosteroides*, and up to 85% losses of colonies of long-lived gorgonians were found [8], [9]. In sandy substrates, the storm buried at least 20% of *Posidonia oceanica* seagrass beds at depths less than 10 m, and seriously damaged and destroyed an unknown amount by abrasion, unearthing and uprooting of plants (T. Alcoverro, personal observation) (Fig. 3), which is particularly significant given the suspected very low recovery rate of this community.

In situ measurements of current speed and sediment transport during the passage of the storm were made along the Blanes submarine canyon at water depths ranging between 300 and 1500 m (Fig. 1) by means of three moorings with sediment traps and current-meters that had been deployed in November 2008. On December 26 and early 27, while waves were impacting the nearby coast, down-canyon current speeds up to 70 cm s^−1^ were recorded in the canyon head (Fig. 2E). Current speed increases following the axis of the canyon were also recorded at 1200 and 1500 m. The strong shoreward winds piled up seawater along the coast, and the southwards flowing Northern Current induced an intense alongshore flow that was captured by the nearby canyon head. In addition, bottom shear stress produced by surface waves alone was enough to resuspend sediments, thus increasing the density of the shelf water and easing it to flow downcanyon. The canyon acted as a major outlet for shelf sediment export, as shown by the high suspended sediment concentrations (up to 28 g m^−3^) recorded at 1200 m 10–20 h after each current speed pulse at 300 m (Fig. 2F).

**Figure 4:**
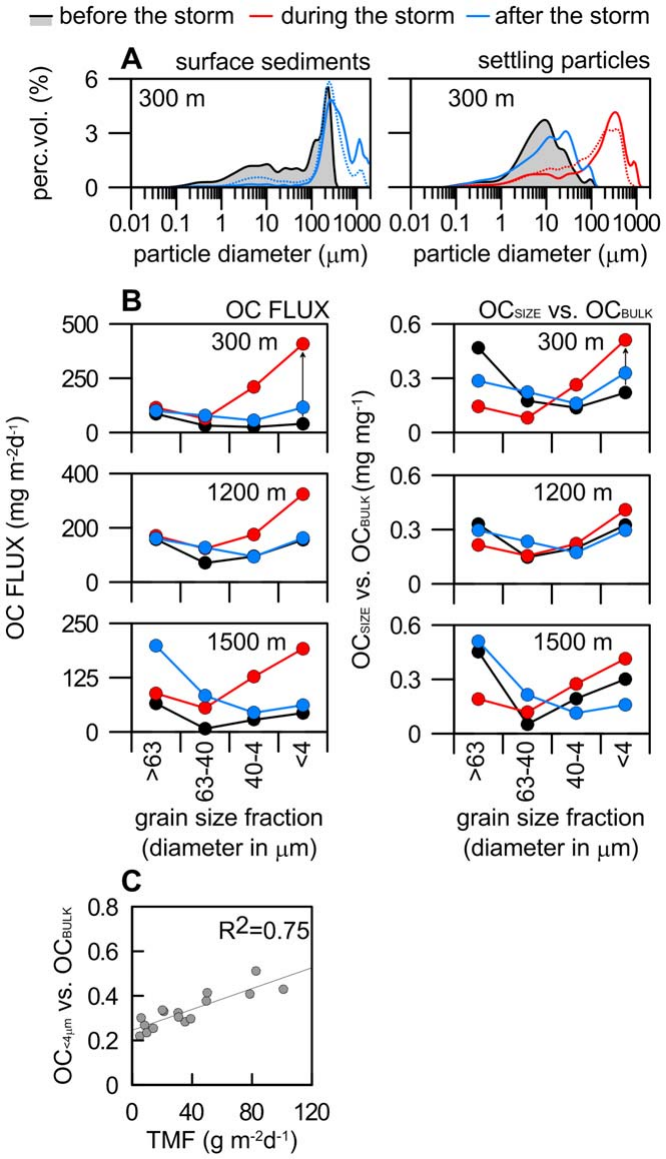
Grain size and geochemical parameters. **A**, Detailed grain size distribution of surface sediments (left) and settling particles (right) at the head of the Blanes canyon before (black line), during (red line) and after (blue line) the storm that hit the Catalan coast on December 26, 2008. The storm triggered a massive arrival of coarse sediments at the canyon head, including sand and carbonate debris as shown by the difference of the HCl decarbonated (dotted line) and bulk grain size distribution (solid line). **B**, OC flux (left) and OC load (right) in each size-fraction of the bulk sample before (black line), during (red line) and after (blue line) the storm. Note the significant increase of the OC load of the fine fraction during the storm. **C**, Relationship between total mass flux (TMF, g m^−2^d^−1^) and OC contained in the fine (<4 µm) fraction. A linear regression of the data implies that hydrodynamic forcing drives both TMF and OC loading of particles in the deep sea.

As a consequence of the wave-current shear stress over the shelf floor large volumes of sand and carbonate debris were supplied into the canyon. This is illustrated by the significant increase in grain size when comparing pre- and post-storm seafloor sediments inside the Blanes canyon (Fig. 4A). Grain size at 300 m water depth changed from unimodal well-sorted fine sand (0.25 mm) in November 2008 to a bimodal distribution (0.3 mm and 1.5 mm) in May 2009. Active winnowing of fine-grained particles due to wave attack might have raised and resuspended shelf sediments, with very coarse sand and biogenic carbonate debris from depths less than 60 m (where the canyon head cuts the shelf) dropped into the canyon to water depths of, at least, 300 m (Fig. 4A). The storm-induced transport of very coarse sand and carbonate debris is also observed on sediments caught by the sediment trap deployed 25 m above the bottom at the same station, with an increase from 2 to 71% of the sand fraction (up to 60 gr sand m^−2^d^−1^) and a similar bimodal distribution (Fig. 4A). Suspended load transport of 1.5 mm sized particles requires a current speed of 160 cm s^−1^ [10] thus indicating that these velocities may have been reached nearby the 300 m water depth station into the canyon. Mechanical breakdown of abraded biological material from the continental shelf during transport might have increased both the amount of exported carbonate debris and the abundance of organic matter in the suspended particle pool.

In addition to mobilizing and dropping coarse shelf particles into the canyon upper reaches, the turbulence created by the storm waves formed a turbid flow that entered the canyon, where it triggered an increase of settling particles, including organic carbon (OC), which was recorded down to 1500 m deep (Fig. 4B). This raises the question about the extent to which coastal storms affect carbon budgets. To determine the quantity and the source of particulate OC exported during the storm event we have measured the OC concentration and stable carbon isotopic signature in several grain size fractions of settling particles and sediments (see the Methods section). We find that, during the storm, most of the exported OC was contained within the finest (less than 4 µm) size fraction (Fig. 4B). This illustrates how large amounts of fine particles with high OC-content can be remobilized in a matter of hours due to a coastal storm. Once suspended, this large pool of OC associated to the fine fraction can travel long distances, thus being transported more efficiently to the deep than previously thought (Fig. 4C).

The absence of macrodetritus in sediment trap samples may suggest the lack of direct transport from macrophyte (algae or seagrasses) communities destroyed by the storm to the depth. However, post-storm observations between Tossa and Blanes (Fig. 1) confirmed an extensive reworking of sediments within seagrass meadows in the inner shelf, which jointly with the high OC contents stored in the associate sediments [11], reinforces the hypothesis of a connection between shallow water processes and the deep-sea.

The linear relationship between OC content and grain size is pretty well known [12], [13], as it is the association between terrestrial OC and the fine mineral fraction of sediments [14]. Recent studies have demonstrated that tropical cyclones [15] and continental erosion [16] increase the riverine transport and burial of recently fixed terrestrial OC in the ocean. Contrary to what could have been expected, and in spite of river flooding (Fig. 2d), we find that such atmospheric drivers are also efficient at transferring suspended OC of marine origin. Indeed the mineral-bound OC advected offshore was dominantly marine-derived, whereas the contribution of terrestrial OC was mostly contained in the silt (4–63 µm) fraction (Table S1). This indicates that organic-mineral interactions occur in the coastal environment, in line with the views of [17] and [18].

Our observation that marine OC is well preserved and tightly associated with clay particles in the shelf implies that sediment resuspension due to wave attack and subsequent lateral advection is highly relevant for the supply of marine OC to the deep ecosystem. Our results indicate that OC loading and the nature of particles settling in the deep-sea relates to hydrodynamic forcing in the coastal sea, and that atmospherically-driven injection of tons of marine OC-rich clay particles into the deep-sea may represent a highly relevant component of the global ocean carbon cycle (Fig. 4c). Assuming that the fine particles caught by the sediment traps are representative of the OC load of the turbid plume, and that each downcanyon current speed pulse at the canyon head spreads suspended matter as far as 1200 m deep at least (Fig. 2F), we estimate that OC exported through the Blanes canyon during this single storm accounted for 5,563 t OC or 2,384 t OC d^−1^. This is higher than the average daily export of 1,873 t OC d^−1^ due to Dense Shelf Water Cascading (DSWC) in the nearby Gulf of Lion, a type of marine current driven exclusively by seawater density contrast [19], but two orders of magnitude lower than the total OC exported during the whole DSWC event, as it lasted for 40 days whereas the storm duration was less than 3 days. The flood in the Tordera River generated by the storm delivered less than 40 t OC to the shelf, which represented less than 1% of the total exported OC.

These results have profound implications for the current understanding of deep-sea biology. In spite of their catastrophic effect on the coastal communities (or, more exactly, thanks to it), storms of high magnitude largely contribute to the sustainment of the deep ecosystem through the episodic supply of large volumes of marine OC mostly along submarine canyons. This adds a new view to the current understanding on the impacts that climate-driven phenomena may have on deep-sea ecosystems, and consequently, on their living resources [19], [20].

How human-induced climate change might alter large, storm-triggering winds becomes then critical also for the deep-sea ecosystem relying on the arrival of food inputs from shallower ocean compartments. Several studies give evidence of a decrease in the total number of cyclones in the Western Mediterranean Sea under climate change integrations (see review in [21]). However, wind and wave intensity during storm events is expected to increase [22], [23]. This means that, for a given depth, the amount and frequency in the arrival of OC without oxidation due to coastal storms will increase, and also the probability of OC to be isolated from the atmosphere. This process boosts the sequestration of OC to the deep and thus, reinforces the negative feedback on the global warming related to the increase of CO_2_. It also counteracts the negative effect of global warming on the reduction of OC sequestration due to less frequent and less intense DSWC and the shallowing of the winter mixed layer depth in most of the Mediterranean Basin [19], [24].

## Materials and Methods

### Instrumented moorings and sediment samples

Instrumented moorings were deployed along the axis of the Blanes submarine canyon at 300, 1200, and 1500 m depth during winter 2008–09 (black dots in Fig. 1). Each mooring was equipped with a PPS3 Technicap sequential sediment trap (0.125 m^2^ opening, 12 collecting cups) at 25 m above the bottom and an Aanderaa RCM7/9 current meter at 23 m above the bottom. Sediment trap sampling intervals were set at 15 days except for the station at 1200 m when a 7/8-day interval was applied. Sediment trap intervals as referred to Fig. 4 correspond to 7–15 or 23 November 2008 (before the storm), 25 December 2008–10 January 2009 (during the storm), and 10–26 January 2009 (after the storm). The current meter at 1200 m depth was further equipped with an optical backscatter sensor model 3612A from Aanderaa Data Instruments. Turbidity values were converted into suspended sediment concentration following the calibration curve given in [25]. Sampling intervals were set at 30 minutes. Undisturbed bottom sediment samples were collected with an 8-tube multicorer system at the same stations in November 2008 and February/May 2009. The top cm of one multicorer tube per station was used for isotopic and grain-size analyses. No specific permits were required for the described field studies. The sediment sampling locations are not privately-owned or protected in any way.

### Analytical methods

Sediment trap sample processing is described in detail by [26]. One split of each sediment sample was fractionated into 4 size fractions: <4 µm, 4–40 µm, 40–63 µm, and >63 µm. The sample was suspended and disaggregated with sodium polyphosphate for 2 h and sequentially passed through 63 and 40 µm stainless steel sieves to collect sand and coarse-silt sized material. Material passing the 40 µm sieve was centrifuged 7 minutes at 1000 rpm to separate the fine-silt (deposit) from the clay-sized (supernatant) fraction. This step was repeated twice. The suspension containing the <4 µm fraction was recovered with 15 minute centrifugation at 5000 rpm. A Coulter LS 230 Laser Particle Size Analyzer was used to compare the size distribution and adjust the centrifugation times. Each fraction was then freeze-dried and ground to a fine powder. Samples for OC analysis were decarbonated using repeated additions of 25% HCl with 60°C drying steps in between until no effervescence was observed. OC and TN contents, and ∂^13^C were measured on a Flash 1112 EA interfaced to a Delta C Finnigan MAT isotope ratio mass spectrometer at the Scientific-Technical Services of the University of Barcelona. Uncertainties on ∂^13^C were lower than 0.15‰ as determined from routine replicate measurements. Grain size analyses on the bulk sample were conducted on the laser particle size analyzer after organic matter oxidation with 10% H_2_O_2_, and with and without decarbonation with 25% HCl.

### Oceanographic and fluvial data

Meteorological data (wind gust, wind direction, rain) were obtained from the Espolla, Palafrugell and Malgrat de Mar weather stations (red dots in Fig. 1) from Servei Meteorològic de Catalunya and MeteoPalafrugell. Wave measurements were obtained from a scalar DATAWELL Waverider Buoy belonging to the Xarxa d'Instruments Oceanogràfics i Meteorològics (XIOM) from the Generalitat de Catalunya (Blanes buoy) and from a Triaxys directional Wave Buoy belonging to Puertos del Estado from the Spanish Government (Palamós buoy) (blue dots in Fig. 1). The Tordera river discharge was obtained from the Fogars de Tordera gauging station from the Agencia Catalana de l'Aigua (yellow dot in Fig. 1).

### 26^th^ of December 2008 storm modelling

Meteorological data during the storm was simulated by the Weather Research and Forecasting model with the Advanced Research dynamical solver (WRF-ARW) version 3.1 [27]. This atmospheric model is a coupling Limited Area Model (LAM), which can be run on mesoscale areas. The grid domain covers the European region in a Lambert conformal conic projection, focusing on the Western Mediterranean Basin. The global ERA-Interim reanalysis from ECMWF with a spatial resolution of ∼0.7° [28] was used as boundary condition. The dynamic downscaling was configured with 40 log-distributed vertical levels, ∼15 km horizontal resolution, and 60-second time step for numerical stability. The outputs of hourly wind fields at 10 m height were used as forcings in the wave model. Waves propagate through the Mediterranean Sea transporting the accumulated energy obtained from the wind. The generation and propagation of the wind waves was simulated using the third generation model WaveWatch III, version 2.22 [29]. The hindcast on the whole Mediterranean Sea was computed on a grid domain with a spatial resolution of 1/8°. The influence of the North Atlantic Ocean in the Western Mediterranean Basin has been considered by Atlantic wave spectra in the input boundaries. Wave growth uses source terms to account for wind input, non-linear wave-wave interactions and whitecapping. Effects of depth-induced refraction are also considered in the propagation model. The minimum propagation time-step used for the computation was 60 seconds and the spectral resolution covers 72 regularly spaced directions. Frequencies extend from 0.03679 Hz with 25 frequency steps. The numerical results of surface winds and significant wave height were validated by comparison with observations: hourly records of metocean buoys were used to validate time series whereas altimeter observations provided a useful tool for spatial validation.

## Supporting Information

Figure S1 **Long term records of significant wave height.** Significant wave height (Hs) in the Palamós and Blanes buoys. See location of the buoys in Fig. 1.The threshold value above 4.3 m, corresponding to storm categories 4 (severe storm) and 5 (extreme storm) of [6], is also shown (dotted line).
(TIF)

Table S1 **Isotopic composition and OC/N ratio of settling particles along the Blanes canyon.** δ^13^C and OC/TN atomic ratio in each size-fraction before, during and after the storm that hit the Catalan coast on December 26, 2008. A δ^13^C value close to 21‰ corresponds to organic matter from marine primary production [30], whereas δ^13^C of terrestrial OC at the outlet of the Tordera river averages 28.5‰ (n = 28). The increase of OC flux and OC loading during the storm (Fig. 3), together with the marine character of the OC, suggest the transfer of fine particles derived from primary production in the shelf. For reference, unfractionated sediments had δ^13^C values from −23.3 to −25.5‰, precluding any interpretation on OC sources. Note that OC/TN ratio of fine particles during the storm also points to a fresh nature of OC [31]. n.d. means no data available due to lack of sediment samples for δ^13^C analyses.
(DOC)

Video S1 **Storm animation.** Simulation of wind field (left, m s^−1^) and significant wave height (right, m) during the December 26, 2008 severe storm. Wave buoys used to validate time series are also shown (blue dots, see Fig. 1). Details on the atmospheric model are provided in the Supporting Methods section.
(MP4)

## References

[1] Lionello P, Bhend J, Buzzi A, Della-Marta PM, Krichak SO (2006). Cyclones in the Mediterranean region: Climatology and effects on the environment.. Dev Earth Environ Sci.

[2] Canals M, Lastras G, Urgeles R, Casamor JL, Mienert J (2004). Slope failure dynamics and impacts from seafloor and shallow sub-seafloor geophysical data: case studies from the COSTA project.. Mar Geol.

[3] Lastras G, Canals M, Amblas D, Lavoie C, Church I (2011). Understanding sediment dynamics of two large submarine valleys from seafloor data: Blanes and La Fonera canyons, northwestern Mediterranean Sea.. Mar Geol.

[4] Ferré B, Guizien K, Durrieu de Madron X, Palanques A, Guillén J (2006). Fine-grained sediment dynamics during a strong storm event in the inner-shelf of the Gulf of Lion (NW Mediterranean).. Cont Shelf Res.

[5] Guillén J, Palanques A, Puig P, Durrieu de Madron X, Nyffeler F (2006). Sediment dynamics during wet and dry storm events on the Têt inner shelf (SW Gulf of Lions).. Mar Geol.

[6] Mendoza ET, Jiménez J (2009). Regional vulnerability analysis of Catalan beaches to storms.. P I Civil Eng Mar En.

[7] Bolanos R, Jordà G, Cateura J, Lopez J, Puigdefabregas J (2009). The XIOM: 20 years of a regional coastal observatory in the Spanish Catalan coast.. J Marine Syst.

[8] Garcia-Rubies A, Mateo MA, Coma R, Hereu B, Zabala M (2009). Preliminary assessment of the impact of an extreme storm on Catalan Mediterranean shallow benthic communities.. Plinius Conf Abstr.

[9] Navarro L, Ballesteros E, Linares C, Hereu B (2011). Spatial and temporal variability on deep-water algal assemblages in the Northwestern Mediterranean: insights into the effects of an exceptional storm.. Estuar Coast Shelf Sci.

[10] Li MZ, Amos CL (2001). SEDTRANS96: the upgraded and better calibrated sediment-transport model for continental shelves.. Comput Geosci.

[11] Kennedy H, Beggins J, Duarte CM, Fourqurean JW, Holmer M (2010). Seagrass sediments as a global carbon sink: Isotopic constraints.. Global Biogeochem Cy.

[12] Mayer LM (1994). Surface area control of organic carbon accumulation in continental shelf sediments.. Geochim Cosmochim Ac.

[13] Hedges JI, Keil RG (1995). Sedimentary organic matter preservation: an assessment and speculative synthesis.. Mar Chem.

[14] Keil RG, Tsamakis E, Giddings JC, Hedges JI (1998). Biochemical distributions among size-classes of modern marine sediments.. Geochim Cosmochim Ac.

[15] Hilton RG, Galy A, Hovius N, Chen MC, Horng MJ (2008). Tropical-cyclone-driven erosion of the terrestrial biosphere from mountains.. Nature Geosci.

[16] Galy V, France-Lanord C, Beyssac O, Faure P, Kudrass H (2007). Efficient organic carbon burial in the Bengal fan sustained by the Himalayan erosional system.. Nature.

[17] Dickens AF, Baldock JA, Smernik RJ, Wakeham SG, Arnarson TS (2006). Solid-state ^13^C NMR analysis of size and density fractions of marine sediments: Insight into organic carbon sources and preservation mechanisms.. Geochim Cosmochim Ac.

[18] Arnarson TA, Keil RG (2007). Changes in organic matter-mineral interactions for marine sediments with varying oxygen exposure times.. Geochim Cosmochim Ac.

[19] Canals M, Puig P, Heussner S, Durrieu de Madron X, Palanques A (2006). Flushing submarine canyons.. Nature.

[20] Company JB, Puig P, Sardà F, Palanques A, Latasa M (2008). Climate influence on deep sea populations.. PLoS ONE.

[21] Ulbrich U, Leckebusch GC, Pinto JC (2009). Extra-tropical cyclones in the present and future climate: a review.. Theor Appl Climatol.

[22] Young IR, Zieger S, Babanin AV (2011). Global Trends in Wind Speed and Wave Height.. Science.

[23] Marcos M, Jordà G, Gomis D, Pérez B (2011). Changes in storm surges in southern Europe from a regional model under climate change scenarios.. Global Planet Change.

[24] Somot S, Sevault F, Déqué M (2006). Transient climate change scenario simulation of the Mediterranean Sea for the twenty-first century using a high-resolution ocean circulation model.. Clim Dynam.

[25] Guillén J, Palanques A, Puig P, Durrieu de Madron X, Nyffeler F (2000). Field calibration of optical sensors for measuring suspended sediment concentration in the Western Mediterranean.. Sci Mar.

[26] Heussner S, Ratti C, Carbonne J (1990). The PPS 3 time-series sediment trap and the trap sample processing techniques used during the ECOMARGE experiment.. Cont Shelf Res.

[27] Skamarock WC, Klemp JB, Dudhia J, Gill DO, Barker DM (2008). A description of the advanced research WRF version 3..

[28] Dee DP, Uppala S (2009). Variational bias correction of satellite radiance data in the ERA-Interim reanalysis.. Quart J R Meteorol Soc.

[29] Tolman HL (2002). User manual and system documentation of WAVEWATCH-III version 2.22..

[30] Fry B, Sherr EB (1984). ^13^C measurements as indicators of carbon flow in marine and freshwater ecosystems.. Contrib Mar Sci.

[31] Hedges JI, Keil RG, Benner R (1997). What happens to terrestrial organic matter in the ocean?. Org Geochem.

