# Supplementary figures and images for: Impacts on the Deep-Sea Ecosystem by a Severe Coastal Storm
Source: PLoS One. 2012 Jan 25;7(1):e30395. doi: 10.1371/journal.pone.0030395 (PMC3266243; doi:10.1371/journal.pone.0030395)

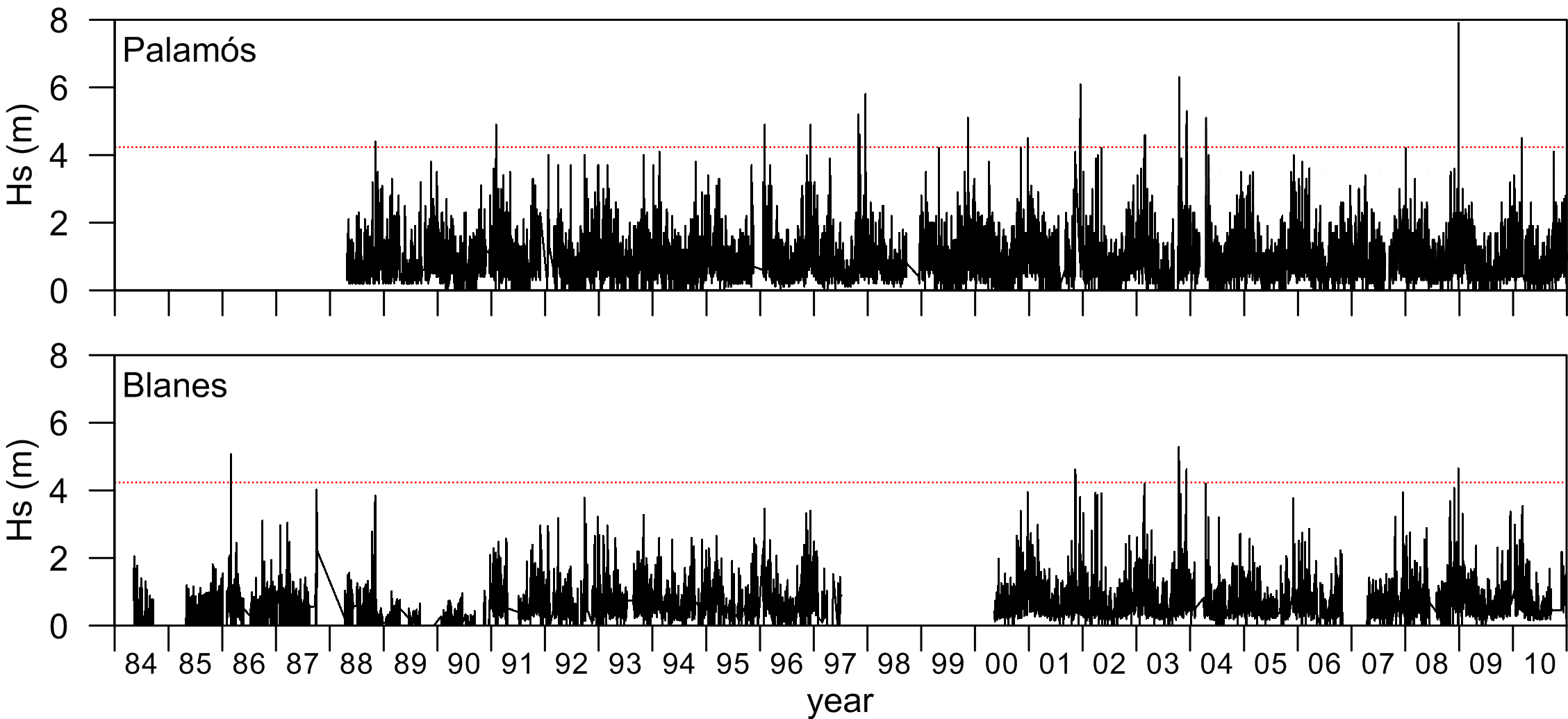

Supplement: Figure S1 — Long term records of significant wave height. Significant wave height (Hs) in the Palamós and Blanes buoys. See location of the buoys in Fig. 1.The threshold value above 4.3 m, corresponding to storm categories 4 (severe storm) and 5 (extreme storm) of [6], is also shown (dotted line). (TIF) [file pone.0030395.s001.tif]
