# Supplementary material for: Impacts on the Deep-Sea Ecosystem by a Severe Coastal Storm
Source: PLoS One. 2012 Jan 25;7(1):e30395. doi: 10.1371/journal.pone.0030395 (PMC3266243; doi:10.1371/journal.pone.0030395)
Supplement: Table S1 — Isotopic composition and OC/N ratio of settling particles along the Blanes canyon. δ13C and OC/TN atomic ratio in each size-fraction before, during and after the storm that hit the Catalan coast on December 26, 2008. A δ13C value close to 21‰ corresponds to organic matter from marine primary production [30], whereas δ13C of terrestrial OC at the outlet of the Tordera river averages 28.5‰ (n = 28). The increase of OC flux and OC loading during the storm (Fig. 3), together with the marine character of the OC, suggest the transfer of fine particles derived from primary production in the shelf. For reference, unfractionated sediments had δ13C values from −23.3 to −25.5‰, precluding any interpretation on OC sources. Note that OC/TN ratio of fine particles during the storm also points to a fresh nature of OC [31]. n.d. means no data available due to lack of sediment samples for δ13C analyses. (DOC) [file pone.0030395.s002.doc]

**Table S1**

| **Station** | **Grain size**  **fraction (μm)** | **δ^13^C** | **OC/TN** |
| --- | --- | --- | --- |
| **Before the storm** | | | |
| BC300 | >63 μm | -23.33 | 7.0 |
|  | 63-40 μm | n.d. | 9.6 |
|  | 40-4 μm | -23.38 | 8.9 |
|  | <4 μm | -23.17 | 8.8 |
| BC1200 | >63 μm | n.d. | 9.7 |
|  | 63-40 μm | n.d. | 11.0 |
|  | 40-4 μm | -26.49 | 8.9 |
|  | <4 μm | -24.89 | 10.1 |
| BC1500 | >63 μm | n.d. | 10.1 |
|  | 63-40 μm | n.d. | 11.0 |
|  | 40-4 μm | -23.64 | 9.5 |
|  | <4 μm | -23.90 | 9.0 |
| **During the storm** | | | |
| BC300 | >63 μm | -22.97 | 9.2 |
|  | 63-40 μm | -23.91 | 9.2 |
|  | 40-4 μm | -27.06 | 9.3 |
|  | <4 μm | -23.88 | 8.3 |
| BC1200 | >63 μm | -23.95 | 10.0 |
|  | 63-40 μm | -24.98 | 12.5 |
|  | 40-4 μm | -27.23 | 8.9 |
|  | <4 μm | -23.00 | 8.4 |
| BC1500 | >63 μm | -23.92 | 9.6 |
|  | 63-40 μm | -25.52 | 10.4 |
|  | 40-4 μm | -27.58 | 9.6 |
|  | <4 μm | -22.95 | 8.6 |
| **After the storm** | | | |
| BC300 | >63 μm | -24.47 | 15.1 |
|  | 63-40 μm | -27.64 | 17.1 |
|  | 40-4 μm | -25.10 | 10.2 |
|  | <4 μm | -23.09 | 8.8 |
| BC1200 | >63 μm | -24.89 | 12.8 |
|  | 63-40 μm | -26.26 | 12.1 |
|  | 40-4 μm | -26.27 | 9.0 |
|  | <4 μm | -24.26 | 8.4 |
| BC1500 | >63 μm | -25.15 | 9.7 |
|  | 63-40 μm | -28.43 | 15.3 |
|  | 40-4 μm | -23.27 | 7.9 |
|  | <4 μm | -23.17 | 7.5 |
